# Wilde on Diseases of the Ear

**Published:** 1853-12

**Authors:** 


*Wilde on diseases of the Ear.* Philadelphia: Blanchard & Lee,

Of all the branches of surgery that relating to diseases of the ear has probably been most neglected, and it is for the purpose of rescuing these diseases from the hands of quacks and charlatans, that Mr. Wilde has sought in his book to make them a more general object of attention and study. The work before us is full and ample, giving evidence of being written by one whose object is to describe accurately,; and those descriptions are all based upon careful observation and study. We notice particularly that he condemns the common habit of pouring laudanum or stimulating liquids into the ear, when it happens to ache. Without taking up a routine treatment, the way should be to examine the patient, see what is the *cause* of the pain, and if possible remove it. Glycerine, too, which has been so much vaunted, meets with but little favor at his hands. In "nervous deafness", *irrespective of organic lesion of the brain*, he says he has nothing to offer but an ear trumpet and a recommendation not to quack.—There is one good caution as to prognosis, which it is well to remember, viz. "so long as ottirrhœa is present we never can tell how, when, or where it will end, or what it will lead to."

We can recommend this work as a valuable addition to a medical library. For sale by Cook & Co. M.

